# Heat-stone massage for patients with chronic musculoskeletal pain: a protocol for multicenter randomized controlled trial

**DOI:** 10.3389/fmed.2023.1215858

**Published:** 2023-08-16

**Authors:** Li Li, Yawei Xi, Ying Wang, Yinqiu Gao, Xiaoying Lv, Shu Liu, Guangjing Yang, Jingjing Qian, Xiaofang Yang, Nardeen Ayad, Jiayan Zhou, Ya Xuan Sun, Jin Liu, Jinlin Li, Guang Chen

**Affiliations:** ^1^Guang’anmen Hospital, China Academy of Chinese Medicine, Beijing, China; ^2^Institute of Basic Research in Clinical Medicine, China Academy of Chinese Medical Sciences, Beijing, China; ^3^The First Affiliated Hospital, Henan University of Chinese Medicine, Zhengzhou, Henan, China; ^4^Chongqing Traditional Chinese Medicine Hospital, Chongqing, China; ^5^Massachusetts General Hospital, Harvard Medical School, Harvard University, Boston, MA, United States; ^6^Department of Medicine, Stanford University School of Medicine, Stanford, CA, United States; ^7^Harvard T. H. Chan School of Public Health, Harvard University, Boston, MA, United States; ^8^Johns Hopkins University School of Medicine, Baltimore, MD, United States; ^9^John F. Kennedy School of Government, Harvard University, Cambridge, MA, United States; ^10^Broad Institute of MIT and Harvard, Cambridge, MA, United States

**Keywords:** massage, complementary medicine, chronic pain, randomized clinical trial, protocol

## Abstract

**Introduction:**

Chronic musculoskeletal pain bothers the quality of life for approximately 1.71 billion people worldwide. Although pharmacological therapies play an important role in controlling chronic pain, overuse of opioids, persistent or recurrent symptoms, and pain-related disability burden still need to be addressed. Heat-stone massage is using the heated stone to stimulate muscles and ligaments followed by massage for relax, which can potentially treat the chronic musculoskeletal pain. To determine the efficacy and safety of heat-stone massage for patients with chronic musculoskeletal pain is needed.

**Methods and analysis:**

This multicenter, 2-arm, randomized, positive drug-controlled trial will include a total of 120 patients with chronic musculoskeletal pain. The intervention group will receive a 2 week heat-stone massage, 3 times per week, whereas the control group will receive the flurbiprofen plaster twice per day for 2 weeks. The primary end point is the change in Global Pain Scale from baseline to the end of the 2 week intervention. The secondary outcomes include the pain severity (Numerical Rating Scale), pain acceptance (Chronic Pain Acceptance Questionnaire), self-management (Health Education Impact Questionnaire), self-efficacy (Pain Self-Efficacy Questionnaire), anxiety and depression (Hospital Anxiety and Depression Scale), quality of life (Short Form-36). The intention-to-treat dataset will be used for analysis.

**Discussion:**

The pain management remains the research topic that patients always pay close attention to. This will be the first randomized clinical trial to evaluate whether heat-stone massage, a non-pharmacological therapy, is effective in the chronic musculoskeletal pain management. The results will provide evidence for new option of daily practice.

**Clinical trial registration:**

World Health Organization Chinese Clinical Trial Registry [ChiCTR2200065654; https://www.chictr.org.cn/showproj.html?proj=185403]; International Traditional Medicine Clinical Trial Registry [ITMCTR2022000104; http://itmctr.ccebtcm.org.cn/en-US/Home/ProjectView?pid=51776b6f-77b8-4811-9b5a-a0fec10f2cee].

## Introduction

Chronic musculoskeletal pain, affecting bones, muscles, ligaments, tendons, and joints, bothers the quality of life for approximately 1.71 billion people worldwide, according to the report of World Health Organization (1). Virtually everyone, over the course of life span, has some form of musculoskeletal pain (2). Because of its negative impact on the locomotor system, chronic musculoskeletal pain particularly the low back pain is the leading contributor to disability in 160 countries (3, 4). Moreover, the chronic pain can significantly increase the overuse of drug consumption, higher frequency of sick leave, earlier retirement from work, and can reduce the well-being levels and society participation capacity (5).

Pharmacological therapies include simple analgesics, opioids, and adjuvant, which were originally proposed by WHO known as the pain ladder system, based on the management guideline of musculoskeletal pain (2). Although these therapies play an important role in chronic pain management, their efficacy can be sometimes limited, and there is safety concern. A systematic review summarized the common problems in musculoskeletal pain care--overuse of imaging, overuse of surgery, overuse of opioids, and failure to provide education and advice (6). Despite multiple first-line therapies recommended in chronic musculoskeletal pain guidelines, many patients still suffer from persistent or recurrent symptoms, leading to the trend that the burden disability caused by chronic pain in patients with chronic musculoskeletal disorders had increased by 46% since 1990 until 2010 in 21 regions worldwide (7). Although the guideline suggests non-pharmacological therapies such as physical modalities, cryotherapy, heat therapy, transcutaneous, and acupuncture, researchers and primary care providers still highly recommends that apply manual therapy only as an adjunct to other evidence-based treatment, indicating there is lack of evidence for these non-pharmacological therapies.

Heat-stone massage is using the heated stone to stimulate the muscles, ligaments, and tendons, followed by massage for relaxation. In the framework of Chinese medicine theory, chronic musculoskeletal pain can be regarded as local stagnancy and lack of nourishment in the related meridians; meanwhile, heat can be used to nourish the meridians, stone can be used to stimulate the acupoints to smooth the stagnancy, and massage can relax the local tissue after the heat-stone stimulations to enhance the pain relief. One clinical trial with 56 participants enrolled showed that focal massage with high force significantly lower visual analog scale compared with low force (mean difference of −1.33) (8). Another clinical trial with a total of 78 participants showed that moxibustion-*cum*-massage significantly reduced the pain severity assessed by brief pain inventory compared to control group of no treatment (mean difference of −0.76) (9). Our *unpublished* pilot study showed that heat-stone massage reduced the pain assessed by global pain scale (GPS) by a mean of 4. We hypothesis that this novel heat-stone massage would reduce pain level in patients with chronic musculoskeletal pain. This randomized controlled trial is aimed to investigate the efficacy and safety of heat-stone massage for patients with chronic musculoskeletal pain.

## Methods and analysis

### Study design

This study is a randomized, positive drug controlled, clinical trial, which will include a total of 120 patients with chronic musculoskeletal pain. Potential participants who are diagnosed by chronic musculoskeletal pain based on categories in International Classification of Diseases version 11 (ICD-11) for both inpatient and outpatient settings will be screened for eligibility criteria. All included patients will be randomly assigned into either the heat-stone massage group or positive drug control (flurbiprofen plaster) group at a 1:1 ratio. The primary outcome is the pain level assessed by global pain scale. This protocol is in accordance with the Standard Protocol Items: Recommendations for Interventional Trials reporting guideline (SPIRIT) (10).

### Participants recruitment

**Figure 1:**
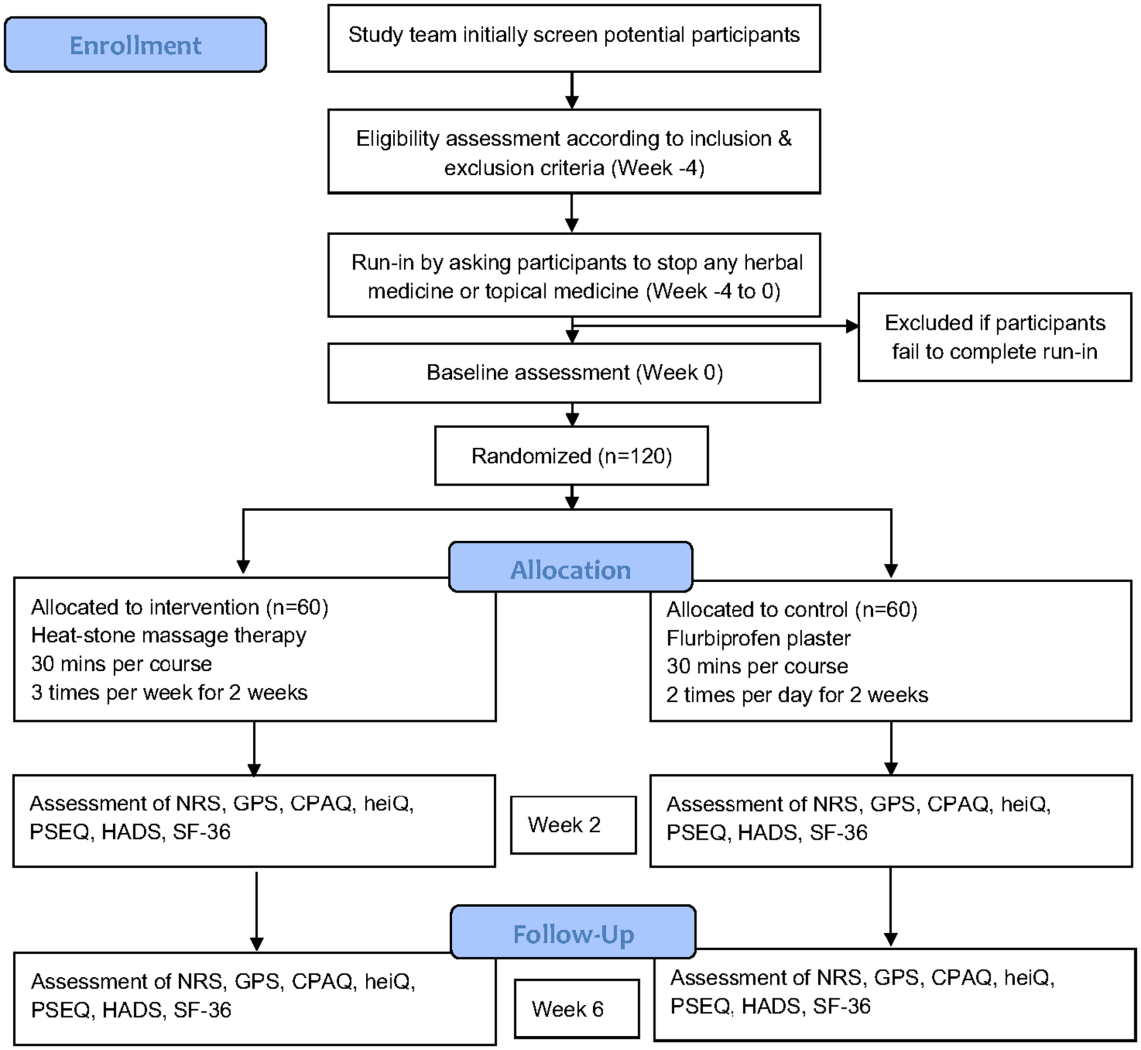
Flow diagram of the study protocol. NRS, Numerical rating scale; GPS, Global pain scale; CPAQ, Chronic pain acceptance questionnaire; heiQ, Health Education Impact Questionnaire; PSEQ, Pain Self-Efficacy Questionnaire; HADS, Hospital anxiety and depression scale; SF-36, Short Form-36.

This clinical trial is being conducted in Guang’anmen Hospital, China Academy of Chinese Medical Sciences (Beijing, China), the First Affiliated Hospital of Henan University of Chinese Medicine (Henan, China), and Chongqing Traditional Chinese Medicine Hospital (Chongqing, China). The study has been recruiting patients since November 2022. We expect the study can be complete in September 2024. Participants are screened at outpatient pain clinic or in the hospital ward. Research staff will visit potential participants who meet the criteria and give them a comprehensive introduction to our study aim and protocol and invite them to join the study. All participants will sign the informed consent form before formal enrollment in our study. The study workflow chart is shown in Figure 1.

### Inclusion and exclusion criteria

The inclusion criteria of this trial are (1) chronic primary musculoskeletal pain is clinically diagnosed based on the criteria that chronic primary musculoskeletal pain is chronic pain in the muscles, bones, joints or tendons that is characterized by significant emotional distress such as anxiety, anger/frustration or depressed mood or functional disability such as interference in daily life activities and reduced participation in social roles, including fibromyalgia syndrome which is defined as diffuse pain in at least 4 of 5 body regions which is associated with significant emotional distress or functional disability; (2) chronic musculoskeletal pain symptoms more than 3 months; (3) pain severity of Numerical Rating Scale (NRS) pain score between 4 and 6; (4) pain is often related to weather changes, and symptoms can be aggravated after rainy days and fatigue; (5) age between 18 years and 80 years; (6) volunteer to participate in the trial and sign the informed consent.

The exclusion criteria of this trial are (1) acute pain or chronic secondary musculoskeletal pain or chronic cancer related pain or chronic neuropathic pain; (2) history of cardiovascular and cerebrovascular diseases, liver diseases, kidney diseases, blood system diseases, diabetes, or infectious diseases; (3) history of tuberculosis, rheumatoid arthritis, ankylosing spondylitis, gout, or cancer bone metastasis; (4) history of mental disorders or communication dysfunction; (5) history of peripheral nerve perception disorder; (6) pregnant, lactating women or those who have pregnancy plans; (7) surgical plan in the next 3 months; (8) reported allergy to the interventions in this trial; (9) have used pain killers or received other pain treatment within the last 1 week.

### Randomization and blinding

All the included participants will be randomly assigned to the heat-stone therapy group (treatment group) or the flurbiprofen plaster group (control group) with a 1:1 ratio with a block size of 6 (R, version 4.2.0). The statistician in our team (X.L) will produce the randomization number list for 3 trial centers, respectively, using central randomization system at the Clinical Assessment Center of China Academy of Chinese Medical Sciences, then randomization list will be automatically sealed in the system including the treatment assignment in it, which will not be seen by any other research staff. This statistician who oversees the randomization will not participate in any process of data collection or outcome analysis. A study nurse in our team (Y.W.) who is not involved in the randomization number generation or patient recruitment process, will use the randomization sequence in the central randomization system to assign the patients in the outpatient or inpatient centers of the hospital in person. Then the patients will be randomly assigned into the heat-stone massage group or flurbiprofen plaster group. Both the participants and heat-stone massage therapists are aware of the treatment, but the principal investigator of this trial, outcome assessment research staff and data analysis statistician will be blinded to the randomization.

### Baseline assessment

Baseline visit and assessment will be conducted at the inpatient centers in the hospitals. Demographic information (including sex, age, educational background, job), course of the chronic pain, medical history (including complications, smoking, alcohol), and basic tests (including blood pressure, heart rate), will be collected in person. Baseline assessment about the chronic musculoskeletal pain will include severity of the pain (assessed by Numerical Rating Scale, range 0–10, higher scores indicate worse pain), pain-related overall life assessment (measured by Global Pain Scale, range 0–200, higher scores indicate worse influence on life), acceptance of pain (assessed by Chronic Pain Acceptance Questionnaire, range 0–120, higher scores indicate higher pain acceptance), health education and self-management (assessed by the Health Education Impact Questionnaire, range 40–200, higher scores indicate better health self-management), pain-related self-efficacy (assessed by the Pain Self-Efficacy Questionnaire, range 0–60, higher scores indicate better confidence of self-efficacy), anxiety and depression (assessed by the Hospital Anxiety and Depression Scale, range 0–42, higher scores indicate more severe symptoms), and quality of life (assessed by Short Form-36, range 0–100, higher scores indicate better health). All scheduled visits are shown in the Table 1.

**Table 1:** The schedule of planned visits.

Study component	Visit 1	Visit 2	Visit 3	Visit 4	Visit 5	Visit 6	Visit 7	Visit 8
Time	1 d before treatment	First treatment	7 ± 1 d after treatment	14 ± 1 d after treatment	21 ± 1 d after treatment	28 ± 1 d after treatment	35 ± 1 d after treatment	42 ± 1 d after treatment
Informed consent	×	–	–	–	–	–	–	–
Demographic	×	–	–	–	–	–	–	–
Pain duration	×	–	–	–	–	–	–	–
Pain locations	×	–	–	–	–	–	–	–
Pain triggers	×	–	–	–	–	–	–	–
Randomization	×	–	–	–	–	–	–	–
Intervention	–	×	×	×	–	–	–	–
NRS	×	–	×	×	–	–	–	×
GPS	×	–	×	×	–	–	–	×
CPAQ	×	–	×	×	–	–	–	×
heiQ	×	–	×	×	–	–	–	×
PSEQ	×	–	×	×	–	–	–	×
HADS	×	–	×	×	–	–	–	×
SF-36	×	–	×	×	–	–	–	×
Pain killer intake	×	–	×	×	–	–	–	×
Adverse events	–	×	×	×	×	×	×	×

NRS, Numerical rating scale; GPS, Global pain scale; CPAQ, Chronic pain acceptance questionnaire; heiQ, Health Education Impact Questionnaire; PSEQ, Pain Self-Efficacy Questionnaire; HADS, Hospital anxiety and depression scale; SF-36, Short Form-36.

### Interventions and control

**Figure 2:**
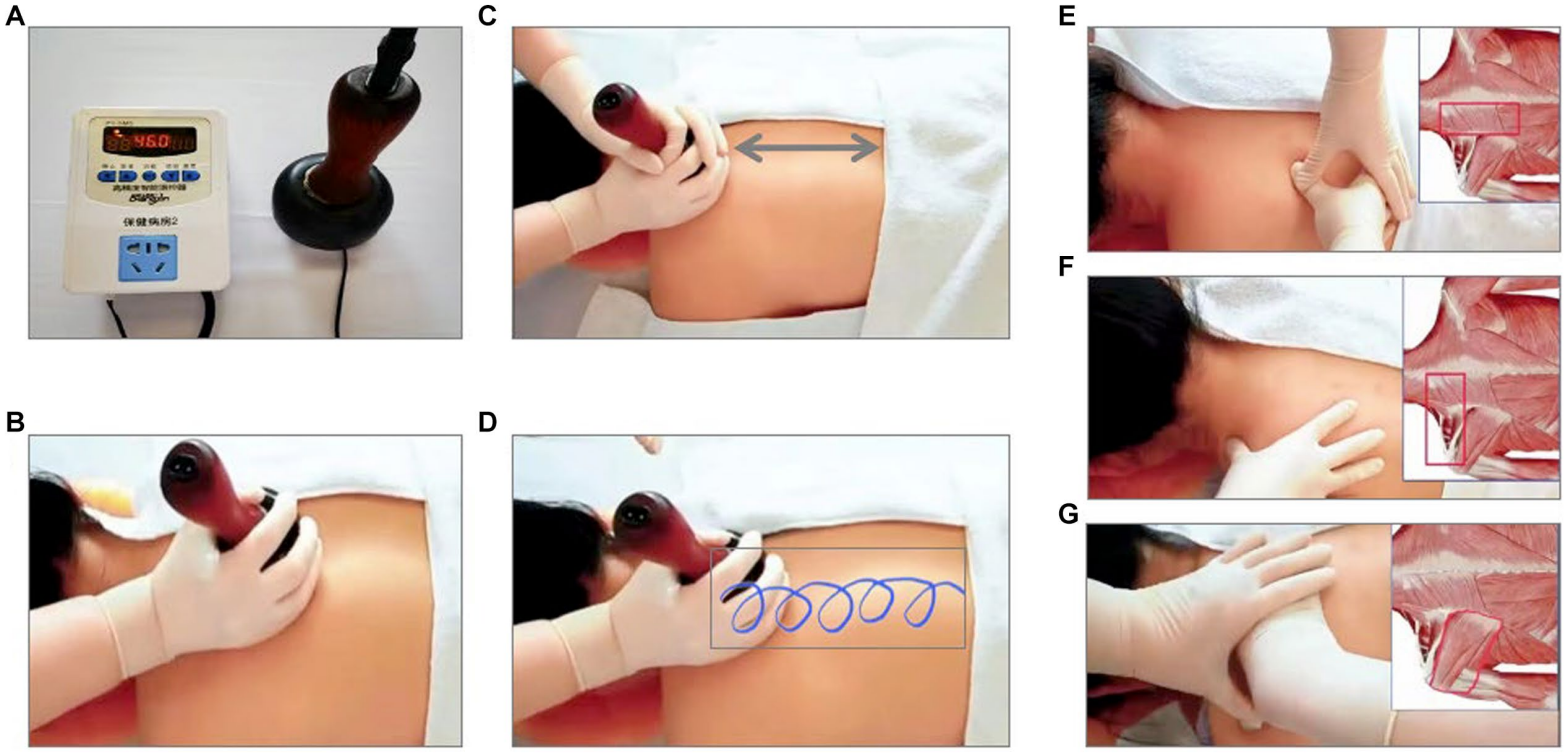
The Standard Procedure of Heat-Stone Massage. **(A)**: heat the stone to 45–50°C; **(B)**: use lubricant on the skin; **(C,D)**: press the heated stone on the skin and move the stone nearby with pressure for 15 min; **(E)**: massage on trapezius, erector spinae, levator scapula, rhomboids major, rhomboids minor for 5 min; **(F)**: massage on trapezius, levator scapula, supraspinatus for 5 min; **(G)**: massage on infraspinatus, teres major, teres minor for 5 min.

After randomization, patient who are enrolled in this trial will get prepared at the operating rooms in the inpatient centers, with the painful parts of the body exposed. The intervention group will receive the heat-stone massage. The heat-stone massage is a standard procedure: (1) use lubricant on the skin where the patients feel painful; (2) heat the stone to 45–50°C and press the heated stone on the skin and move the stone nearby with pressure for 15 min; (3) massage for 15 min. The patients in the heat-stone massage group will receive the standard procedure 3 times per week for 2 weeks. This heat-stone device and massage procedure has already obtained the utility model patent (No. 18576425). All the heat-stone massage providers have been educated in clinical nursing and have been practicing chronic pain management for at least 5 years. The control group will receive the flurbiprofen plaster on the area where patients feel painful, twice per day for 2 weeks. The standard procedure of heat-stone massage is shown in the Figure 2.

### Follow-up visit

The visits will be conducted at the day when patients receive the first treatment, and 7 days, and 14 days after the first treatment, which will be during the intervention duration of 2 weeks. After the end of the intervention, the follow-up visits will be done at day 21, day 28, day 35, and day 42 after the first treatment, which is 1 month follow-up after the intervention.

### Study outcomes and assessment

The primary outcome is the change in the score of Global Pain Scale (GPS, range 0–200, higher scores indicate worse influence on life) from baseline to the end of the 2 week intervention. The GPS is a validated, concise, and easily interpreted score to comprehensively assess the pain, emotions, clinical outcomes, and daily activities (11). The Chinese version of the GPS was also validated in Chinese population (12).

The secondary outcomes include (1) the change in GPS from baseline to the end of 6 week follow-up; (2) the proportion of participants who have clinically significant change in severity of the pain assessed by Numerical Rating Scale (NRS, range 0–10, higher scores indicate worse pain) (13), a change of 20% from baseline to the end of the 2 week intervention is regarded as clinically significant change (14); (3) the change in acceptance of pain assessed by Chronic Pain Acceptance Questionnaire(CPAQ, range 0–120, higher scores indicate higher pain acceptance) from baseline to the end of 2 week intervention (15); (4) the change in health education and self-management assessed by the Health Education Impact Questionnaire (heiQ, range 40–200, higher scores indicate better health self-management) from baseline to the end of 2 week intervention (16); (5) the change in pain-related self-efficacy assessed by the Pain Self-Efficacy Questionnaire (PSEQ, range 0–60, higher scores indicate better confidence of self-efficacy) from baseline to the end of 2 week intervention (17); (6) the change of anxiety and depression assessed by the Hospital Anxiety and Depression Scale (HADS, range 0–42, higher scores indicate more severe symptoms) from baseline to the end of 2 week intervention (18); (7) the change in quality of life assessed by Short Form-36 (SF-36, range 0–100, higher scores indicate better health) from baseline to the end of the 2 week intervention (19).

### Safety and adverse event reporting

We will monitor the adverse events through the trial, that will be achieved by a research board composed by 1 physician, 1 nurse, and 1 statistician. Because the intervention contains heat therapy and pressure on the skin, empyrosis, bruise, and over sweating are likely to occur during the treatment. Any adverse events related to the study intervention will be reported.

### Data collection and data management

Data will be collected through case report forms at each of patients’ visit. A study database will be generated to enter data using EpiData software version 3.1. All the informed consent and case report forms will be locked in the research closet with the access to researchers only at Guang’anmen Hospital, China Academy of Chinese Medical Sciences. All data will be double checked and merged at the end and delivered to the statisticians for analysis. Regarding the quality control, all heat-stone massage providers and outcome assessors at each site will be trained about the standard operation procedure before the start of this trial. The training will include diagnosis of chronic musculoskeletal pain, inclusion and exclusion criteria, operation of the heat-stone, location of the massage points, massage manipulation techniques, the use of the tools for the outcome assessments, and the completion of case report forms. The trial will be monitored by the clinical research center of China Academy of Chinese Medicine.

### Power analysis and sample size calculation

The power analysis and sample size estimation were based on the primary outcome of this trial which is the change in GPS from baseline to the end of the 2 week intervention. One of our pilot studies showed that the mean change in GPS in heat-stone group was 12, while the mean change in GPS in flurbiprofen was 8, with a combined standard deviation of 6.7. With a power of 80%, 2-sided alpha of 0.05, and the assumption that the change is normally distributed, we used the “power twomeans” function in STATA with 1:1 ratio in treatment and control group to get estimated sample size of 46 samples for each group, so that a total of 92 participants are required. Considering the 20% dropout rate, the total sample size of 115 is required. To make it convenient to assign the participants with 2:1:1 ratio to the 3 centers, we took the 120 as the final planned sample size. The sample size was calculated using STATA, version 16.1 (StataCorp, USA).

### Statistical analysis

The Primary analysis will be conducted on the full dataset of all the participants after randomization based on the intention-to-treat principle. Moreover, sensitivity analysis will be conducted between the full dataset and the per protocol dataset. Participants who are not in the per protocol analysis are nonadherence which might be caused by failure to receive the assigned treatment, suffering from severe adverse events, or loss of follow-up.

To check if there is selection bias and confounding factors, we will compare the baseline characteristics of the two groups. Continuous variables will be described as mean (Standard Deviation) for normal distribution and be tested using t test or be described as median (interquartile range) for skewed distribution and be tested using Mann–Whitney test. Categorical variables will be summarized as counts (percentages) and be tested using Chi squared test or Fisher exact test.

The primary outcome of this trial is the change in Global Pain Scale from baseline to the end of the 2 week intervention. The difference of the changes between the two groups will be analyzed using a generalized linear mixed model. The mean and 95% confidence interval will be calculated and estimated. Known confounding factors that may affect the pain severity in chronic musculoskeletal patients include age, sex, job, duration of the chronic pain, and emotional state. Therefore, we will make the subgroup analysis for the variables of age categories (>60 years vs. ≤60 years), sex (female vs. male), job categories, duration of pain, taking these as covariates in the model.

For the secondary outcomes, generalized linear mixed model will be used for continuous variables, and two proportion z test will be used for proportions, whereas the Chi squared test or Fisher exact test will be used for categorical variables.

The primary economic analysis will be the cost-utility analysis over 6 week using quality-adjusted life years (QALYs) calculated from the SF-36. The mixed-effect linear regression model will be used, adjusting the confounding factors including age, sex, job, and site of recruitment.

All statistical tests will use the statistical significance of 2-sided *p* value of <0.05. All the statistical analysis will be performed using Python, version 3.8.16 (sklearn and statsmodels packages).

## Discussion

This is the first randomized clinical trial to investigate the heat-stone massage as a kind of therapy alone to treat chronic musculoskeletal pain. Currently, no massage therapy has been recommended by any of the chronic musculoskeletal pain management guideline due to lack of evidence. Finding a therapy which is easy to use and has long-term effect with low cost for the management of chronic musculoskeletal pain will change the clinical practice of this chronic pain and will change the life quality of the patients. Our pilot study showed a promising result in terms of long-term symptoms relief, but we need strong evidence to prove this. The results of this multicenter, randomized, clinical trial will provide high-quality evidence, which is promising to change the guideline for chronic pain management in the future.

The strengths of this study include: (1) randomization can reduce the selection bias and control both measured and unmeasured confounding factors; (2) inclusion criteria of chronic musculoskeletal pain and standard procedure of the heat-stone massage increase the external validity and application of the intervention; (3) prespecified subgroup analysis can identify any interaction on the causal pathway between the heat-stone massage and pain relief outcome, which will effect modifiers that are crucial when we interpret the results.

This trial also has limitations. First, this trial is only blinded to principal investigator, outcome assessment staff, and outcome analysis statistician, but not blinded to participants or massage therapists, therefore there might be performance bias. Nevertheless, we design the expectation score to evaluate if the expectation is different between groups after they know what we are receiving. Second, although the trial has follow-up visits of 1-month, longer term effect cannot be known, which somehow limits the interpretation of long-term effect of pain relief. Third, we only recruit patients with the moderate pain assessed by NRS score in the range of 4–6, based on the results of our pilot study showing that patient with NRS score between 4 and 6 benefited from the heat-stone massage. This will restrict the chronic pain population that the results of this study can be generalized to, and patients with more severe pain may not benefit.

This multicenter, randomized, controlled, clinical trial will test the hypothesis that heat-stone massage can improve the pain of adult patients with chronic musculoskeletal pain. The results will provide the evidence of the efficacy and safety of heat-stone massage, which might lead to the update of clinical guidelines and change the routine practice.

## Ethics statement

The Ethics Committee of Guang'anmen Hospital approved the study protocol; the most recent version of the protocol (version 2.0) was approved on 25 January 2022. All participants will sign the informed consent form before formal enrollment in our study.

## Author contributions

LL, YW, YG, YX, and GC: conceptualization and design. XL: central randomization system. YW, JLi, GY, JQ, XY, NA, JZ, YS, and GC: acquisition, analysis, or interpretation of data. LL and GC: drafting of the manuscript. LL: supervision. XL, YS, JLiu, and GC: statistical analysis. All authors: writing-review, revision, and editing. All authors contributed to the article and approved the submitted version.

## Funding

This work is supported by China Academy of Chinese Medical Sciences grant C12021A03002 to LL. The study funder had no role in the design or conduct of this study; data collection, management, analysis, and interpretation; manuscript preparation, review, or approval; or decision to submit the manuscript for publication.

## Conflict of interest

The authors declare that the research was conducted in the absence of any commercial or financial relationships that could be construed as a potential conflict of interest.

## Publisher’s note

All claims expressed in this article are solely those of the authors and do not necessarily represent those of their affiliated organizations, or those of the publisher, the editors and the reviewers. Any product that may be evaluated in this article, or claim that may be made by its manufacturer, is not guaranteed or endorsed by the publisher.
